# The Color Question in a Dental College

**Published:** 1893-03

**Authors:** 


ARTICLE VIII.

### THE COLOR QUESTION IN A DENTAL COLLEGE.

The Faculty of the Dental College of Denver University has decided to resign in a body if U. S. G. Cooper is reinstated as a student of the college by the Board of Trustees, and yesterday notified Chancellor McDowell of its determination. The members of the Faculty are George J. Hartung, Dean of the college; J. M. Norman, W. E. Griswold, A. H. Sawins, Charles F. Dodge, G. A. Dille, J. S. Donaldson, A. C. Watson and J. R. Donaldson, and they all signed their names to a statement of their position, which was sent to the Chancellor yesterday afternoon.

The Faculty met yesterday to consider the case, and was unanimous in its conclusions. The status of the matter from its point of view is summarized as follows:

U. S. G. Cooper applied for admission on November 5th. He was told that the time for entering expired October 10th; that if he entered it could be but for a partial course, and that three full courses would have to be taken to entitle him to a graduating examination Cooper was satisfied and entered for the infirmary course only. A reduction was made in the fee and he was given a matriculation card on payment of fifty dollars

Three or four days afterwards complaints of his presence were made to the Dean, which culminated in written protests, signed by all the dental class except one, against the reception of colored students, but not including in the objection Miss Martha Jordan, a young colored woman who is a member of the class.

The demonstrators reported that several of the lady patients had objected to having Cooper hanging over them and working at their mouths while they were reclining in a dentist's chair, and said they would come no more if he remained. Three students gave notice that they would not attend the school next year if Cooper was there.

The Faculty met on November 12th, and. after full consideration, decided that Cooper's presence was an injury to the college. Dean Hartung had a quiet talk with him and laid the matter frankly before him. Cooper agreed to leave the college, his money was returned and he gave a receipt for it, expressly agreeing that he resigned all claims on the college.

The next the Faculty heard of the case was a letter from Chancellor McDowell, saying that the committee of investigation appointed by the Trustees, which consisted of Governor John Evans and the Chancellor, had decided that Cooper was entitled to reinstatement. The Dean wrote to the Chancellor that the Faculty considered it was entitled to a hearing before any decision was reached. To this the Chancellor replied on Monday to the effect that, Cooper being reinstated, the Trustees would hear the case of the Faculty.

Right here is where the Faculty registers its kick. It claims that if it has the right to admit students, it must have the same right to remove objectionable persons, if the interest of the institution demands it, the students, of course, having the right of appeal, as in this case. The Faculty sets up:

*First*: Cooper was admitted freely and frankly without regard to color.

*Second*: He was entered for only a partial infirmary course.

*Third*: He was requested to withdraw only after consideration.

*Fourth*: The Trustees should hear both Cooper and the Faculty before deciding.

*Fifth*: One side of the case only having been heard, the Faculty cannot consider Cooper as reinstated.

The letter to the Chancellor concludes that the members of the Faculty have been with the college for some time, some since its organization; that they have given their time and labor for a small compensation; that in all their actions they have had the interest of the college at heart, and that if they are now required to do that which in their judgment will be injurious to the college, while they will regret the severance of existing pleasant relations, they beg to tender their resignation at once.

Dean Hartung wrote to J. D. Patterson, of Kansas City, President of the National Association of Dental Faculties, which includes twenty-nine of the principal institutions of the country. Dr. Patterson, in reply, wrote that the Association had never considered the question of the admission of colored students, simply with regard to their color, and probably never would do so. He concludes: "So long as there is a college specially for them (Meharry College), I would send them there, and would not receive them into our class on account of expediency."—*Rocky Mountain News*, Nov. 23, 1892, and *Western Dental Journal.*

